# Clinical characteristics and long-term prognosis of autoimmune pancreatitis with renal lesions

**DOI:** 10.1038/s41598-020-79899-3

**Published:** 2021-01-11

**Authors:** Takuya Ishikawa, Hiroki Kawashima, Eizaburo Ohno, Tadashi Iida, Hirotaka Suzuki, Kota Uetsuki, Jun Yashika, Kenta Yamada, Masakatsu Yoshikawa, Noriaki Gibo, Toshinori Aoki, Kunio Kataoka, Hiroshi Mori, Takeshi Yamamura, Kazuhiro Furukawa, Masanao Nakamura, Yoshiki Hirooka, Mitsuhiro Fujishiro

**Affiliations:** 1 Department of Gastroenterology and Hepatology, Nagoya University Graduate School of Medicine, 65 Tsuruma-cho, Showa-ku, Nagoya, 466-8550 Japan; 2 Department of Endoscopy, Nagoya University Hospital, 65 Tsuruma-cho, Showa-ku, Nagoya, 466-8550 Japan; 3 Department of Gastroenterology and Gastroenterological Oncology, Fujita Health University, 1-98 Dengakugakubo, Toyoake, 470-1192 Japan

**Keywords:** Diseases, Gastroenterology, Nephrology

## Abstract

Autoimmune pancreatitis (AIP) is recognized as the pancreatic manifestation of a systemic IgG4-related disease that can involve various organs, including the kidney. However, renal lesions tend to be overlooked when AIP is diagnosed, and the clinical characteristics and long-term prognosis of AIP with renal lesions are unclear. We retrospectively reviewed 153 patients with AIP diagnosed at our hospital with a median follow-up period of 41 months (interquartile range, 10–86) and classified them into two groups: the KD group (n = 17), with characteristic renal imaging features, and the non-KD group (n = 136). Serum IgG4 levels were significantly higher in the KD group (663 vs. 304.5 mg/dl, *P* = 0.014). No differences were observed between the two groups in terms of steroid treatment [14/17 (82.4%) vs. 112/136 (82.4%), *P* = 1] or in the number of patients who exhibited exacerbation of renal function during treatment [1/17 (5.9%) vs. 8/136 (5.9%), *P* = 1]. However, the cumulative relapse rate was significantly higher in the KD group [61% vs. 21.9% (3 years), *P* < 0.001]. Patients in the KD group had different clinical features with high relapse rates compared with those in the non-KD group, and thus, it is important to confirm the presence of renal lesions in AIP patients.

## Introduction

Autoimmune pancreatitis (AIP) is a unique type of chronic pancreatitis associated with autoimmunity. The concept of AIP was first proposed in 1995^1^, and now it is accepted that there are two distinct subtypes, type 1 and type 2, which have different histological and clinical characteristics^2^. Type 1 AIP, which is the major subtype in Japan and other Asian countries, is recognized as the pancreatic manifestation of a systemic IgG4-related disease (IgG4-RD); this type can involve various organs and systems, such as the biliary system, salivary glands, retroperitoneum, kidneys, thyroids, and lungs, among others^3^. Recently, laminin 511-E8 was found to be a target antigen in AIP^4^. AIP patients with laminin 511-E8 antibodies appear to have distinctive clinical features, and it has been suggested that AIP may comprise different subtypes with different autoantigens. Some authors have reported that renal lesions have been identified by computed tomography (CT) or magnetic resonance imaging (MRI) in 35–38% of patients with AIP^5,6^, and the presence of histological tubulointerstitial nephritis (TIN), with infiltration of IgG4-positive plasma cells, has been reported in patients with AIP^7–9^. The renal lesions associated with AIP are considered to be a specific pathologic feature of IgG4-RD, a disease termed ‘IgG4-related kidney disease (IgG4-RKD)’. The diagnostic criteria of IgG4-RKD^10^ were proposed in Japan in 2011, and since then, the clinical and radiological characteristics of IgG4-RKD have gradually become clear. However, the way in which these renal lesions affect the treatment and long-term prognosis of AIP is still not well understood. Several studies have suggested a relationship between AIP and malignant disease^11–13^, but this is still controversial and no reports have examined the association between AIP with renal lesions and malignancy. Here, we aimed to investigate the clinical characteristics and long-term prognosis of AIP with renal lesions, including its association with malignancy.

## Methods

### Study design

This was a single-center, retrospective study performed at Nagoya University Hospital. This research was conducted in strict compliance with the Declaration of Helsinki and in accordance with the ethical guidelines for medical research involving human subjects. Written informed consent was obtained from each patient or family (if the patient was deceased when obtaining consent), and the study was performed with the approval of the Ethics Committee of Nagoya University Hospital. The content of the research was described on the website of our hospital (approval number: 2019-0519).

### Patients

We retrospectively reviewed 159 patients who were diagnosed with AIP at our hospital between May 2001 and January 2020 and classified them into each type based on the international consensus diagnostic criteria (ICDC)^2^. As the presence of renal lesions in type 2 AIP is rare, we excluded the six cases of type 2 AIP from the included subjects. Finally, a total of 153 patients [134 definitive type 1 (87.6%), 17 probable type 1 (4.6%), 12 not otherwise specified (7.8%)] with a median follow-up period of 41 months [interquartile range (IQR) 10–86] were included in the present study. All these patients received a contrast-enhanced CT scan (CE-CT) at initial diagnosis. We classified the patients into two groups: the KD group (17 patients), which had characteristic imaging findings of IgG4-RKD on CE-CT at the time of AIP diagnosis, and the non-KD group (136 patients) and compared their clinical characteristics and long-term prognosis. The cutoff values of the upper limit of normal serum IgG and IgG4 levels were set at 1700 mg/dl and 135 mg/dl, respectively.

### Evaluation of renal lesions associated with AIP

**Figure 1:**
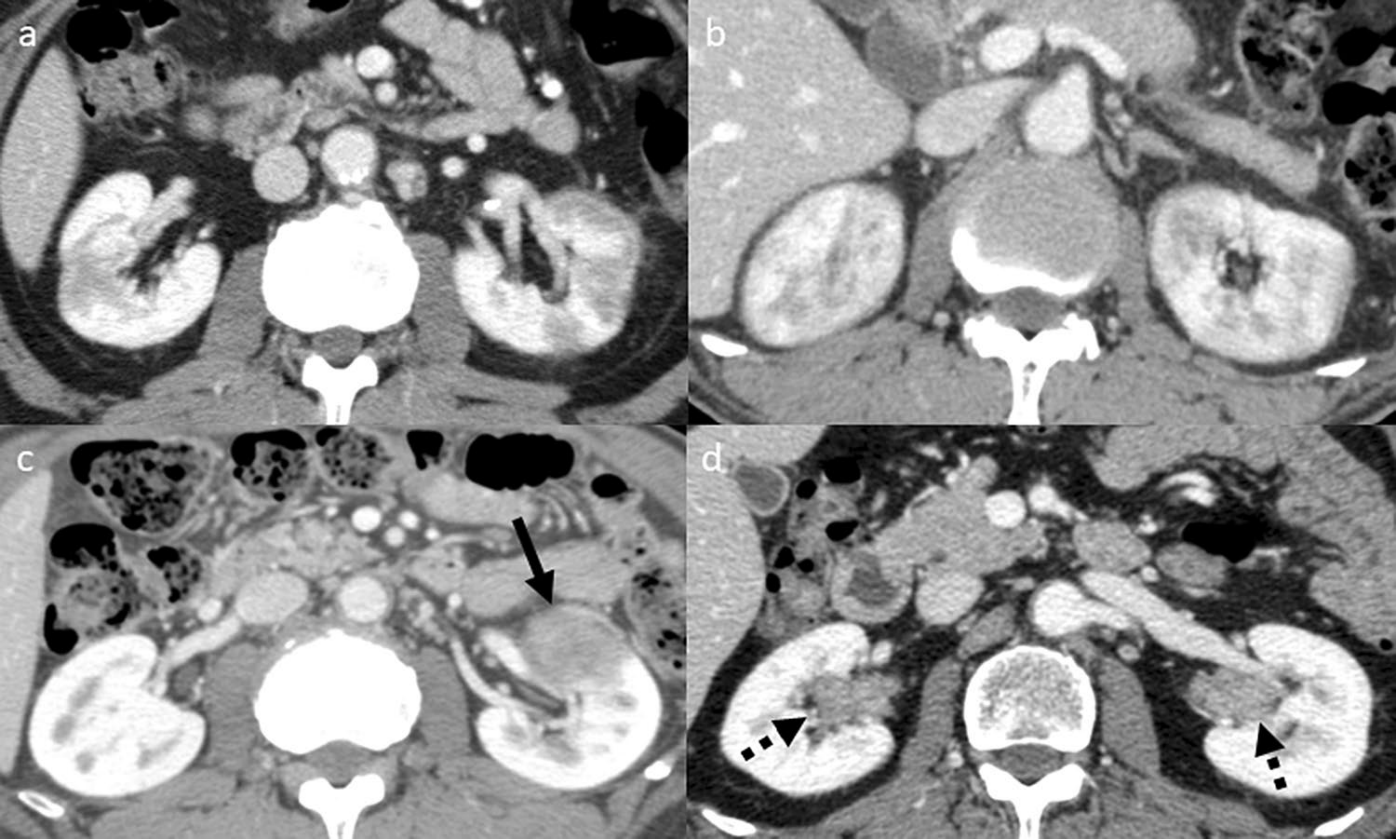
Characteristic findings of IgG4-related kidney disease on contrast-enhanced CT. **(a)** Multiple hypodense lesions. **(b)** Diffuse renal swelling. **(c)** A solitary hypodense lesion (arrow). **(d)** Diffuse thickening of the renal pelvis wall (broken arrows).

Based on the diagnostic criteria for IgG4-RKD^10^ and previous reports^5,6,14,15^, patients who had at least one of the following four imaging features on CE-CT were classified into the KD group: multiple hypodense lesions, diffuse renal swelling, a solitary hypodense lesion or diffuse thickening of the renal pelvis wall (Fig. 1). To evaluate renal function, the estimated glomerular filtration rate (eGFR) was calculated according to the serum creatinine levels in each patient. The eGFRs were classified from G1 to G5 based on the clinical practice guidelines for chronic kidney disease^16^, and the eGFRs at the initial diagnosis of AIP and the changes during the follow-up period were recorded. Those who had eGFR progression of two grades or more were considered to have exacerbation of renal function.

Among the 17 patients in the KD group, 7 patients and 10 patients were diagnosed with definitive IgG4-RKD and possible IgG4-RKD, respectively, according to the diagnostic criteria for IgG4-RKD^10^. Although no patient underwent renal biopsy in our study, all 17 patients underwent endoscopic ultrasound-guided fine needle biopsy of the pancreas, and of these, seven showed dense lymphoplasmacytic infiltration with infiltrating IgG4-positive plasma cells > 10/high power field, which led to the diagnosis of definitive IgG4-RKD.

### Other organ involvement (OOI) apart from renal lesions

Based on the ICDC^2^, in the present study, the following three manifestations were included as OOI in addition to the renal lesions at the onset of AIP: IgG4-related sclerosing cholangitis (IgG4-SC), retroperitoneal fibrosis, and sialadenitis^2^. These manifestations were diagnosed based on histology or typical physical/radiological evidence. IgG4-SC was radiologically defined as the presence of segmental/multiple proximal or proximal and distal bile duct stricture, and patients with only distal bile duct stricture were excluded.

### Treatment and follow-up strategies

Patients were treated with steroids when they had symptomatic disease, including obstructive jaundice and abdominal and/or back pain due to pancreatic involvement or OOI^17^. Prednisolone at an initial dosage of 0.6 mg/kg/day was administered and was subsequently tapered by 5–10 mg every 1 to 2 weeks until the maintenance dosage of 2.5–10 mg/day was reached^18^. The addition of azathioprine was considered for recurrent relapses. Maintenance steroid therapy (MST) was defined as steroid therapy continued at the maintenance dosage for more than 3 years or until relapse. All patients underwent laboratory tests including those for serum creatinine levels as well as CT, magnetic resonance imaging (MRI), or transabdominal ultrasound examination at least every 6 months.

### Long-term prognosis

#### Evaluation of relapse

Remission was defined as the disappearance of clinical symptoms and the resolution of pancreatic and/or extrapancreatic manifestations on imaging studies^19^. With regard to the renal lesions, remission was defined as complete disappearance on CE-CT. The reappearance of symptoms and the development/reappearance of pancreatic manifestations and/or extrapancreatic manifestations, as determined by imaging studies with or without a marked elevation of serum IgG4 levels, were regarded as evidence of relapse^11,18^. Although some patients experienced recurrent relapses during the follow-up period, in the present study, we considered only the first episode of relapse.

#### Association with malignancy

Any cancer that was detected at the time of AIP diagnosis or during follow-up was recorded, and the incidence was compared between the KD group and the non-KD group.

### Statistical analysis

The χ^2^ test and Fisher’s exact test were used to compare categorical parameters, and the Mann–Whitney U test was used to compare continuous variables. Continuous parameters are presented as the median (IQR). Kaplan–Meier curves were drawn to assess the cumulative relapse rates. A multivariate analysis was performed using logistic regression analysis. A *P* value less than 0.05 was considered statistically significant. All statistical analyses were performed using SPSS Statistics 26.0 (SPSS, Inc., Chicago, IL).

## Results

### Radiological findings in the kidneys of patients in the KD group

**Figure 2:**
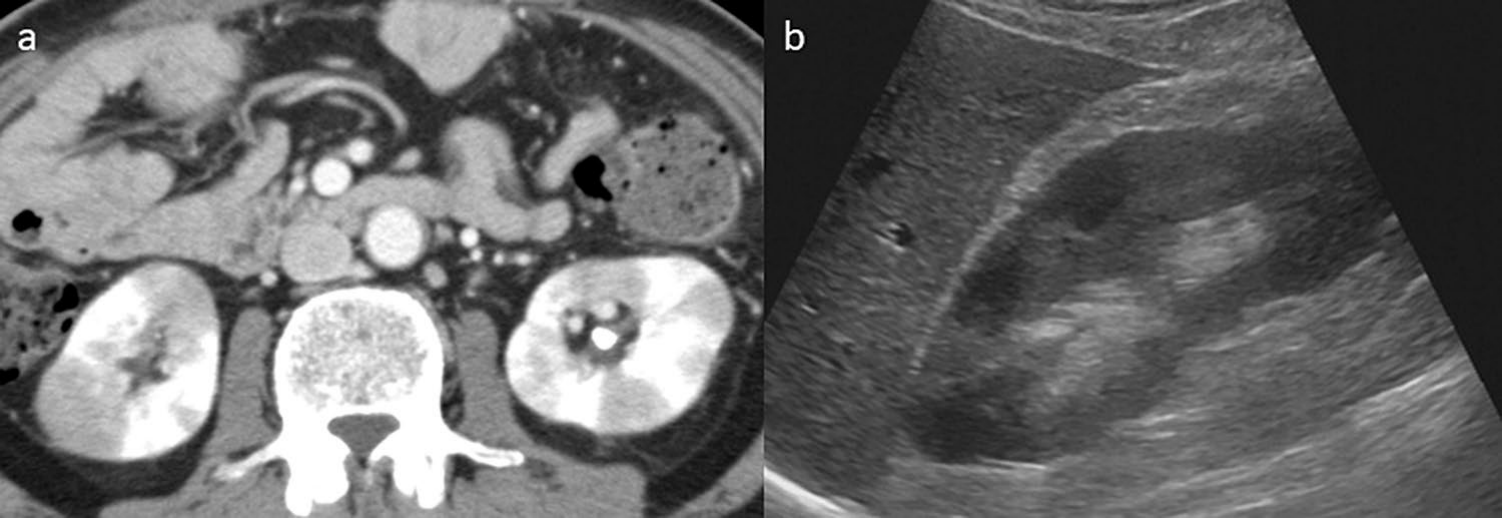
Comparison of the radiological findings of renal lesions between contrast-enhanced CT and transabdominal ultrasound in a patient with AIP. **(a)** A contrast-enhanced CT showing multiple hypodense lesions in both kidneys. **(b)** Transabdominal ultrasound showing multiple ill-defined, non-mass-like areas with decreased echogenicity in the right kidney.

Of the 17 patients in the KD group, 14 (82.4%) had multiple hypodense lesions, 2 (11.8%) had a solitary hypodense lesion, 2 (11.8%) had diffuse thickening of the renal pelvis wall, 1 (5.9%) had diffuse renal swelling on CE-CT, and 2 had multiple findings. All patients underwent transabdominal ultrasound within 1 week after CE-CT; however, abnormalities in the kidneys were detected in only four patients (23.5%) (Fig. 2).

### Differences in clinical characteristics between the KD group and the non-KD group (Table 1)

**Table 1:** Differences between AIP patients with and without renal lesions.

	KD group (n = 17)	Non-KD group (n = 136)	*P*-value
Age, median (IQR)	64 (61–70)	68 (60–74)	0.228
**Sex**			
Male, n (%)	16 (94.1)	113 (83.1)	0.476
Female, n (%)	1 (5.9)	23 (16.9)	
Diabetes (HbA1c > 6.5%), n (%)	11 (64.7)	70 (53)	0.443
CA19-9, median, U/l	13 (6–34)	21 (9–63)	0.232
Serum IgG, median (IQR), mg/dl	2214 (1928–2896)	1747 (1374–2061)	0.001
Serum IgG4, median (IQR), mg/dl	663 (340.5–1036)	304.5 (145.5–618.25)	0.014
Steroid therapy from onset of AIP, n (%)	14 (82.4)	112 (88.9)	1
**Pancreatic parenchymal imaging**			
Diffuse enlargement, n (%)	14 (82.4)	76 (55.9)	0.04
Focal enlargement, n (%)	3 (17.6)	60 (44.1)	
Maintenance steroid therapy, n (%)	10 (71.4)	98 (85.2)	0.243
Number of organs involved at onset of AIP, median (IQR), n	2 (1–2)	0 (0–1)	0.04
IgG4-SC, n (%)	5 (29.4)	28 (20.6)	0.531
Retroperitoneal fibrosis, n (%)	4 (23.5)	13 (9.6)	0.1
Sialadenitis, n (%)	3 (17.6)	9 (6.6)	0.133
At least one disease relapse during the observation, n (%)	10 (58.8)	32 (23.5)	0.004
**Location of relapse, n (%)**			
Pancreas	5 (50)	20 (62.5)	
Biliary system	2 (20)	7 (21.9)	
Salivary gland	1 (10)	2 (6.25)	
Retroperitoneal fibrosis	0	1 (3.13)	
Kidney	1 (10)	1 (3.13)	
Lymphadenopathy	1 (10)	1 (3.13)	

*KD* kidney disease, *IQR* interquartile range, *AIP* autoimmune pancreatitis, *IgG4-SC* IgG4-related sclerosing cholangitis.

Serologically, the median serum IgG and IgG4 levels were significantly higher in the KD group than in the non-KD group [2214 (1928–2896) vs. 1747 (1374–2061) mg/dl, *P* = 0.001, and 663 (IQR 340.5–1036) vs. 304.5 (IQR 145.5–618.25) mg/dl]. In addition, the serum IgG4 level was elevated in all patients in the KD group, and 15 patients (82.4%) had a value of at least 270 mg/dl, which is twice the upper limit of normal. In terms of the pancreatic and extrapancreatic manifestations, diffuse pancreatic enlargement was detected significantly more frequently in the KD group [14/17 (82.4%) vs. 76/135 (55.9%), *P* = 0.04], and the median number of extrapancreatic organs involved was significantly higher in the KD group [2 (IQR 1–2) vs. 0 (IQR 0–1), *P* < 0.001]. No difference was observed between the two groups in the presence or absence of steroid treatment at the initial diagnosis of AIP [14/17 (82.4%) vs. 112/136 (82.4%), *P* = 1].

### Long-term prognosis

#### Renal function (Table 2)

**Table 2:** Differences in renal function between the KD and non-KD groups.

	KD group (n = 17)	Non-KD group (n = 136)	*P*-value
eGFR at initial diagnosis of AIP			
G1, n	1	30	
G2, n	13	80	
G3a, n	1	25	
G3b, n	2	0	
G4, n	0	1	
G5, n	0	0	
G1-G3a, n	15	135	
G3b-5, n	2	1	0.033
Exacerbation of eGFR by two grades or more, n	1	8	1

*eGFR* estimated glomerular filtration rate.

Two patients in the KD group (11.8%) and one patient in the non-KD group (0.7%) (*P* = 0.033) had decreased renal function with eGFRs of 3b or less at the initial diagnosis of AIP. Moreover, one patient (5.9%) and eight patients (5.9%) in the KD and non-KD groups, respectively, had an eGFR exacerbation of two grades or more during the follow-up period, but no statistically significant differences were observed (*P* = 1).

#### Remission and relapse

**Figure 3:**
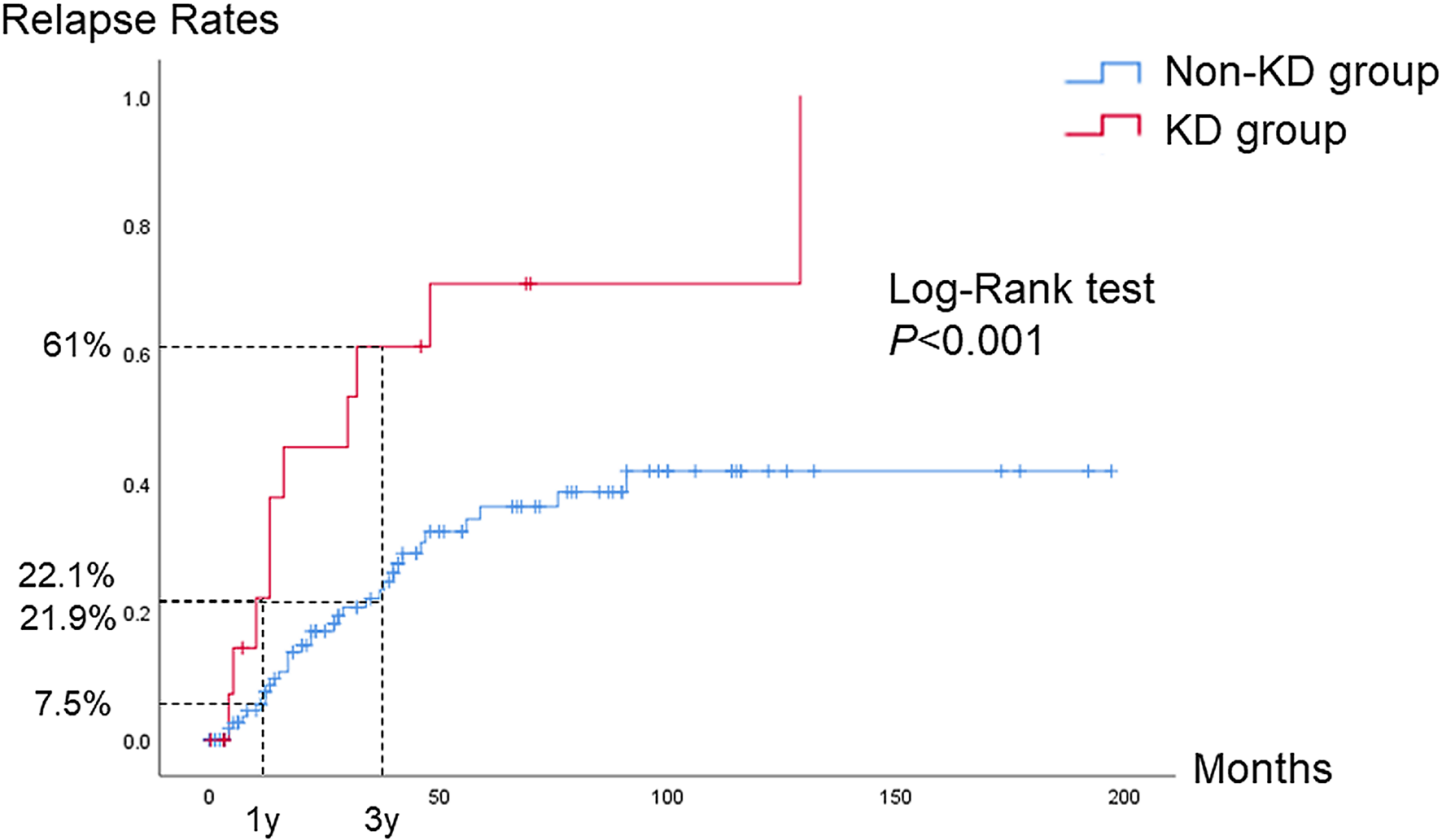
Cumulative relapse rates in the KD group and non-KD group.

Among the 14 patients in the KD group who received steroid therapy, 11 patients achieved remission, as demonstrated by the disappearance of renal lesions on CE-CT. Three patients also showed improvement, but some hypodense areas in the kidney remained. During the median follow-up period of 41 months, a total of ten patients (58.8%) in the KD group and 32 patients (23.5%) in the non-KD group experienced at least one episode of relapse. Of these, five and 12 patients in the KD and non-KD groups, respectively, had relapses in extrapancreatic organs, and one patient in each group had relapses in the kidney (Table 1). The cumulative relapse rates at 1 year and 3 years in the KD and non-KD groups were 22.1% vs. 7.5% and 61% vs. 21.9%, respectively, and the relapse rates were significantly higher in the KD group (log-rank test, *P* < 0.001, Fig. 3). Among the 126 patients who received steroid therapy starting at the onset of AIP, MST was performed in 10 (71.4%) and 98 (85.2%) patients in the KD and non-KD groups, respectively, and this difference was not statistically significant (*P* = 0.243).

#### Multivariate analysis

As the median number of extrapancreatic organs involved was high in the KD group, a multivariate analysis was performed for the clinical features and the relapse rate, which showed a statistically significant difference in the KD group compared with the non-KD group (Table 3). Consequently, IgG4-RKD was identified as an independent factor for serum IgG4 elevation (> 270 mg/dl) and relapse compared with other organ involvement.

**Table 3:** Multivariate analyses for clinical features and relapse rate.

Variables	Serum IgG elevation (> 1700 mg/dl)		Serum IgG4 elevation (> 270 mg/dl)		Diffuse pancreatic enlargement		Relapse	
	Odds ratio 95% CI	*P* value	Odds ratio 95% CI	*P* value	Odds ratio 95% CI	*P* value	Odds ratio 95% CI	*P* value
IgG4-RKD	3.338 0.88–12.64	0.076	5.818 1.20–28.12	0.029	3.255 0.86–12.22	0.08	3.871 1.29–11.60	0.016
IgG4-SC	2.145 0.88–5.23	0.093	1.909 0.80–4.54	0.143	2.192 0.92–5.20	0.075	3.418 1.45–8.02	0.005
Retroperitoneal fibrosis	3.196 0.83–12.22	0.09	0.596 0.18–1.90	0.383	1.039 0.34–3.14	0.946	2.040 0.66–6.22	0.211
Sialadenitis	2.227 0.53–9.33	0.273	6.129 0.73–51.35	0.095	2.164 0.54–8.64	0.275	2.172 0.58–8.08	0.248

*IgG4-SC* IgG4-related sclerosing cholangitis, *IgG4-RKD* IgG4-related kidney disease, *CI* confidence interval.

### Association with malignancy

Of the 153 patients with AIP included in the present study, 14 patients (9.1%) were diagnosed with a malignancy. The detailed characteristics of the malignancies associated with AIP are shown in Table 4. Lung cancer was the most common type of malignancy and was found in 4 patients, followed by gastric cancer in 3 patients, prostate cancer in 2 patients, esophageal cancer in 2 patients, pancreatic cancer in 2 patients, and ovarian cancer in 1 patient. Five patients were diagnosed within 1 year of their AIP diagnosis, and 3 patients were diagnosed with a malignancy at the same time as AIP. Although no significant difference was found in the incidence of malignancies between the 2 groups, all 14 patients with malignancies belonged to the non-KD group, whereas none of the patients in the KD group developed malignancies (*P* = 0.178).

**Table 4:** Characteristics of 14 patients with malignancies.

Patient	Age at onset of AIP	Sex	Type of cancer	Period from the onset of AIP to malignancy (months)	Presence of renal lesions at onset of AIP	OOI at onset of AIP	Steroid therapy from onset of AIP
1	66	M	Lung	153	N	NA	Y
2	77	M	Gastric	15	N	Sialadenitis	Y
3	69	M	Lung	5	N	RF	N
4	77	M	Prostate	27	N	NA	N
5	57	M	Lung	11	N	NA	Y
6	65	M	Prostate	95	N	IgG4-SC Sialadenitis	N
7	76	M	Esophageal	0	N	NA	N
8	55	M	Lung	45	N	NA	Y
9	72	M	Pancreatic	17	N	IgG4-SC	Y
10	64	M	Esophageal	51	N	NA	Y
11	75	M	Gastric	29	N	NA	Y
12	78	M	Gastric	0	N	NA	N
13	53	F	Ovarian	0	N	NA	N
14	75	M	Pancreatic	22	N	IgG4-SC	Y

*AIP* autoimmune pancreatitis, *OOI *other organ involvement, *RF* retroperitoneal fibrosis, *IgG4-SC* IgG4-related sclerosing cholangitis.

## Discussion

In the present study, we compared the clinical characteristics and long-term prognosis of AIP with and without renal lesions, and we found significant differences not only in the clinical characteristics but also in the long-term prognosis.

IgG4-RKD was first reported in 2004^20^ as an extrapancreatic manifestation of AIP. Since then, AIP has been recognized as a pancreatic manifestation of IgG4-related systemic diseases. Accordingly, IgG4-RKD was also classified as an IgG4-related systemic disease, and individual diagnostic criteria for IgG4-RKD were proposed in 2011. Approximately one-fourth to one-third of AIP patients are reported to have IgG4-RKD^5,21,22^. However, although experts recognize that AIP may be associated with renal involvement, in general, gastroenterologists often do not pay much attention to the kidneys when they diagnose AIP. One of the reasons for this is that although progression of IgG4-RKD may lead to renal dysfunction and renal failure, in most patients diagnosed with AIP, renal function remains normal or mildly compromised, even if renal involvement is present. In a retrospective study that evaluated 62 type 1 AIP patients^23^, eight patients showed kidney involvement at the time of AIP diagnosis, but abnormal creatinine values were found in only two patients with mild elevation. In the present study, two patients (11.8%) in the KD group had decreased renal function, with a mild decrease in eGFR of G3b. Therefore, when diagnosing AIP, it is important to assess images for the presence of renal lesions. The possibility of renal lesions is high and requires attention especially in patients with elevated IgG4.

CT is the most recommended radiographic imaging method for IgG4-RKD^10^. In general, CE-CT is needed to make a correct diagnosis. In the present study, 14 of 17 patients (82.4%) in the KD group had multiple hypodense lesions in the kidney on CE-CT. These findings are thought to be specific for IgG4-RKD^6,24,25^, and thus, we should be cautious so that they are not overlooked. Other findings of a solitary hypodense lesion or diffuse thickening of the renal pelvis wall are relatively rare manifestations of IgG4-RKD^14^, but these findings require differentiation from malignant tumors, such as renal cancer or ureteropelvic cancer. In the present study, two patients had these characteristics and will need careful follow-up. It has been reported that ultrasonographic findings of IgG4-RKD are not specific and that ultrasound is less sensitive than CT with regard to lesion detection^26^. Indeed, in the present study, abnormal ultrasonographic findings were detected in only 4 of 17 patients (23.5%) in the KD group. However, the findings of ill-defined, non-mass-like areas with decreased echogenicity are unique features, and once these findings are observed, they can support the diagnosis of patients with suspected AIP.

The response of IgG4-RKD to steroids is considered to be as good as that of other IgG4-related diseases. In a retrospective study that investigated 34 patients with IgG4-RKD^15^, 32 patients were treated with oral steroids, and in 15 patients who underwent follow-up imaging examinations, the number and size of the renal lesions decreased after treatment. In a retrospective study^10^ of 38 IgG4-RKD patients treated with steroids, 35 patients had a good response. However, two patients did not experience improvements in their serum creatinine levels despite steroid therapy, and one patient eventually required hemodialysis. It was suggested that the delay in introducing steroid therapy may have been the cause of treatment failure. In the present study, 82.4% of patients were treated with steroids starting at the time of diagnosis, and only one patient had worsening renal function during follow-up. That patient had a decreased eGFR of G3b before the initiation of steroid therapy. No significant difference was observed in the number of patients who had exacerbation of renal function between the two groups during the follow-up period, and initiation of steroid therapy at an early stage may have contributed to the preservation of renal function.

According to previous studies^3,18^, 30–50% of patients with type 1 AIP experience relapse. However, most risk factors for relapse remain poorly understood. A recent prospective Japanese multicenter study^27^ showed that MST leads to beneficial outcomes after remission. Several previous studies have also shown that the presence of IgG4-SC at initial diagnosis is a predictor of relapse^11,28,29^. In the present study, the cumulative relapse rates were significantly higher in the KD group than in the non-KD group (22.1% vs. 7.5% in one year and 61% vs. 21.9% in 3 years, respectively, *P* < 0.001). No differences in MST implementation or in the prevalence of IgG4-SC were found between the 2 groups, and the presence of renal lesions might be another risk factor for relapse in AIP patients. In the present study, the median number of extrapancreatic organs involved was high in the KD group. Kuruma et al*.*^14^ also reported that the prevalence of extrapancreatic lesions other than renal lesions was significantly higher in AIP patients with IgG4-RKD after evaluating a total of 92 AIP patients including 13 patients with IgG4-RKD. Therefore, we performed a multivariate analysis of other organ involvement, and IgG4-RKD was still identified as an independent factor for relapse in addition to IgG4-SC.

Regarding the association with malignancy, 14 patients (9.1%) in the present study were diagnosed with a malignancy. Interestingly, all 14 patients belonged to the non-KD group, and none of the patients in the KD group developed a malignancy during the study period, although this difference was not statistically significant (*P* = 0.178). AIP patients with laminin 511-E8 antibodies have been reported to have distinctive clinical features, and the frequency of malignancies in patients with these antibodies is significantly lower than that in patients without these antibodies. In addition, laminin is known to have multiple subfamilies, and one such subfamily, laminin 521, was detected only in AIP patients with IgG4-RKD^4^. Although renal involvement was found in only one of 51 AIP patients included in that study, their results suggest that the organ specificity and the clinical features of IgG4-related diseases may differ depending on the subfamily of laminin autoantibodies present in the patients. Further investigation will be needed to clarify these relationships.

The present study has several limitations. First, this was a single-center retrospective study. Second, the diagnosis of renal lesions was based on imaging, and none of the patients underwent renal biopsy. Therefore, IgG4-RKD without typical radiological findings might have been missed. However, the indication for renal biopsy should be carefully determined given its risks, including bleeding, and we believe that the diagnostic process we used accurately reflects real-world practice. Third, of the 17 patients diagnosed with IgG4-RKD in the present study, 11 patients had diabetes and 9 patients were being treated with antihypertensive medication at the time of AIP diagnosis. It is difficult to rule out the possibility that they were the causes of nephropathy in these patients. However, only the patients who showed renal radiological findings characteristics of IgG4-RKD were included in the KD-group, and as a result, we believe that we could minimize the possibility of renal involvement from other causes. Finally, as all patients were admitted to the Department of Gastroenterology, precise laboratory tests for kidney disease, such as measurement of complement 3 or 4 levels, which are reportedly associated with IgG4-RKD^30,31^, as well as other detailed urinary tests were not performed in most patients. Further prospective studies are warranted to evaluate these values and to validate the differences in long-term prognosis between the two groups.

In conclusion, all patients in the KD group had elevated serum IgG4 levels that were significantly higher than those in the non-KD group, and it is necessary to consider that AIP with higher IgG4 levels may be accompanied by renal lesions. The relapse rate was significantly higher in the KD group than in the non-KD group, and tailored maintenance therapy with a higher dosage or longer duration may need to be considered. However, the risk of developing a malignancy appears to be low in these patients, and early therapeutic intervention may contribute to the preservation of renal function.

## Data Availability

The datasets generated and analyzed during the current study are available from the corresponding author on reasonable request.
